# Cross-talk between autophagy and apoptosis regulates testicular injury/recovery induced by cadmium via PI3K with mTOR-independent pathway

**DOI:** 10.1038/s41419-020-2246-1

**Published:** 2020-01-22

**Authors:** Mei Wang, Xiao-fei Wang, Ya-min Li, Na Chen, Yan Fan, Wen-kai Huang, Shi-fu Hu, Meng Rao, Yuan-zhen Zhang, Ping Su

**Affiliations:** 1 Institute of Reproductive Health, Tongji Medical College, Huazhong University of Science and Technology, Wuhan, 430030 Hubei P.R. China; 2 Department of Obstetrics and Gynecology/Reproductive Medicine Center, Zhongnan Hospital, Wuhan University, Wuhan, 430071 Hubei P.R. China; 3 Harvard Reproductive Endocrine Science Center and Reproductive Endocrine Unit of the Department of Medicine, Massachusetts General Hospital, Harvard Medical School, Boston, MA 02114 USA; 4 Reproductive Medicine Center, Tongji Medical College, Huazhong University of Science and Technology, Wuhan, 430030 Hubei P.R. China; 5 Department of Reproduction and Genetics, The First Affiliated Hospital of Kunming Medical University, Kunming, 650000 Yunnan P.R. China

**Keywords:** Experimental models of disease, Macroautophagy

## Abstract

Autophagy and apoptosis are two major modes of cell death. A balanced interplay between both is vital for phagocytic clearance of apoptotic testicular cells. Here, generating a SD rats model-treated with cadmium (Cd) to mimic environmental exposure on human, we show that autophagy and apoptosis present synchronous change trends in Cd-induced testicular injury/self-recovery. Further, the cross-talk of autophagy and apoptosis is investigated in four testicular cell lines (GC-1/GC-2/TM3/TM4 cells) respectively. Results reveal that Cd-exposure for five consecutive weeks induces reproductive toxicity in male rats. After one cycle of spermatogenesis within 8 weeks without Cd, toxic effects are ameliorated significantly. In vitro, we find that PI3K inhibitor 3-MA regulates apoptosis by inhibiting autophagy with mTOR-independent pathway in Cd-treated testicular cells. Conclusively, cross-talk between autophagy and apoptosis regulates testicular injury/recovery induced by Cd via PI3K with mTOR-independent pathway.

## Introduction

Cell apoptosis and autophagy are two major morphologically distinctive forms of programmed cell death (PCD), which plays a significant role in the development and control of male reproductive functions^1^. some studies indicate that autophagy constitutes a stress adaptation to avoid cell death (and suppresses apoptosis), while other research manifests that it constitutes an alternative cell-death pathway^2^, and even the autophagic degradation is a newly defined mechanism of triggering cell death^3^. Given that autophagy plays different roles under various physiological or pathological conditions^4^, it is generally believed that autophagy is a double-edged sword^5^. Cadmium (Cd) is a major industrial and environmental toxicant^6^, which does harm to human health through contaminated foods, water and air^7^. In fact, increasing research indicates that environmental exposure to Cd is related to poor semen quality and male infertility^8^. Previously, the studies of Cd-induced damage to male testes focus on high-dose acute Cd exposure, which impairs the blood-testis barrier^9^ and leydig cells (LCs) regeneration^10^, causing a large amount of germ cell death^11^. Nonetheless, no study to date has investigated the cross-talk between apoptosis and autophagy in testicular injury/self-repair induced by low-dose continuous Cd exposure.

Here, we generate a SD rats model to mimic environmental Cd exposure on human male and explore the role of autophagy and apoptosis in Cd-induced testicular injury/self-recovery. In vitro, PI3K inhibitor 3-methyladenine (3-MA), well known as an autophagy inhibitor^12,13^, is used to further investigate the cross-talk of autophagy and apoptosis in four testicular cell lines (GC-1 cells: mouse spermatogonial cells; GC-2 cells: mouse spermatocyte cells; TM3 cells: mouse leydig cells; TM4 cells: mouse Sertoli cells) respectively. This research explores the underlying molecular mechanism of Cd-induced testicular injury, and for the first time, examines whether autophagy occurs in low-dose continuous Cd-induced testicular injury and self-recovery. More importantly, we verify the cross-talk between autophagy and apoptosis and the role of mammalian target of rapamycin (mTOR) signaling in vivo and vitro. Thus, this study not only provides novel evidence supporting that PI3K mediates interplay between autophagy and apoptosis in testicular cells, but also contributes to the development of new pharmaceutical therapies for male infertility.

## Results

### Cd impaired reproductive function of adult male SD rats, and testes had a certain self-recovery ability

Adult male SD rats were treated with different concentration of Cd (0, 0.2, 0.4, 0.8 mg/kg) for different lengths of time (1–5 weeks), following 8 weeks’ self-recovery period (Supplementary Fig. 1A). Body weights were gained more slowly in the Cd-treated groups (Supplementary Fig. 1B). Five weeks’ 0.8 mg/kg Cd exposure significantly decreased organ coefficients of testis and prostate. After the self-recovery period, the organ coefficients of the testis, liver and spleen recovered (Supplementary Fig. 1C, D).

**Fig. 1:**
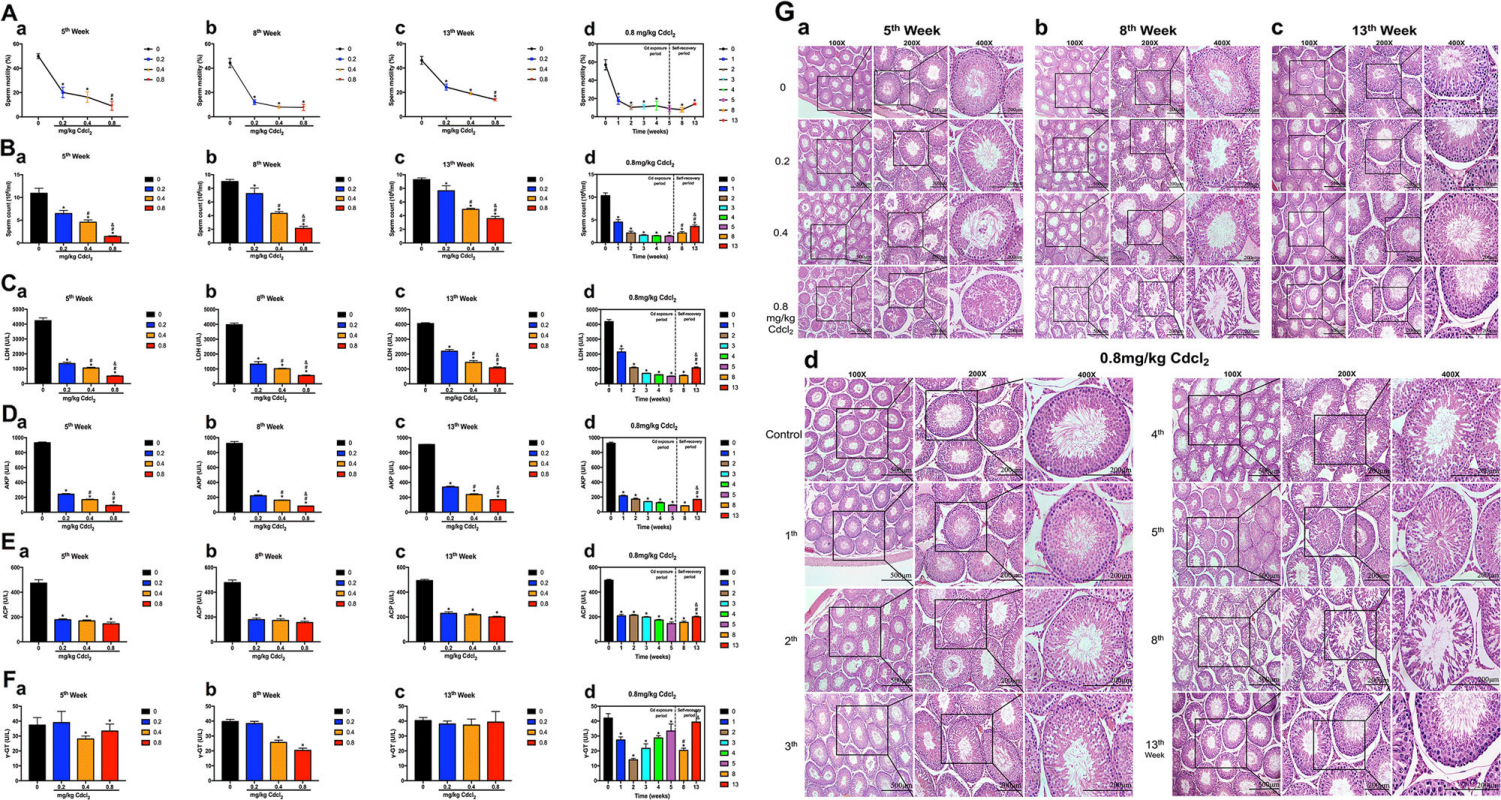
Reproductive function of adult male rats was impaired during the Cd exposure period and recovered during the self-recovery period. **a** Sperm motility. **b** Sperm count. **c**–**f** Testicular cell marker enzyme activity. **g** Histological results of the testis in rats. Original magnification, 100×, 200×, and 400×. When it comes to the comparisons of 5th, 8th, 13th week in 0.8 mg/kg Cd group in **d**, the data of 8th, 13th week is re-used in **b**, **c**.*P < 0.05 compared with the control. ^#^*P* < 0.05 compared with 5th week. ^&^*P* < 0.05 compared with 8th week.

Significantly, Cd reduced sperm motility and counts in a time-dose dependent manner. After the self-repair period, sperm counts were apparently increased at 13th week compared with that at 5th, 8th week; while there was no obvious recovery of sperm motility (Fig. 1a, b).

Cd decreased the activity of the germ cell marker enzymes LDH, AKP and the Sertoli cell marker enzymes ACP in a time-dose dependent manner (Fig. 1c–e). After self-repair period, the activity of LDH and AKP recovered at 13th week (Fig. 1c, d). Another Sertoli cell marker enzyme γ-GT was decreased to the lowest level at 2nd week, gradually recovered at 3rd–5th week, and significantly decreased at 8th week and returned to normal levels at 13th week (Fig. 1e, f).

Cd resulted in testicular morphometric injury. At the same time points, the structure of seminiferous tubules presented slight loose in 0.2 mg/kg Cd group, while severe vacuolization in 0.8 mg/kg Cd group, even that spermatogonia and spermatocytes, were separated from the spermatogenic epithelium (Fig. 1g(a-c)). At the same dose (0.8 mg/kg) group, the damage of the seminiferous tubules was progressively aggravated from 1st to 8th week, and markedly recovered at 13th week (Fig. 1g(d)).

Above results suggested that low-dose persistent Cd exposure induced reproductive toxicity, and testis had a certain self-recovery ability.

### Autophagy and apoptosis presented synchronous change trends in Cd-induced testicular injury/self-recovery

**Fig. 2:**
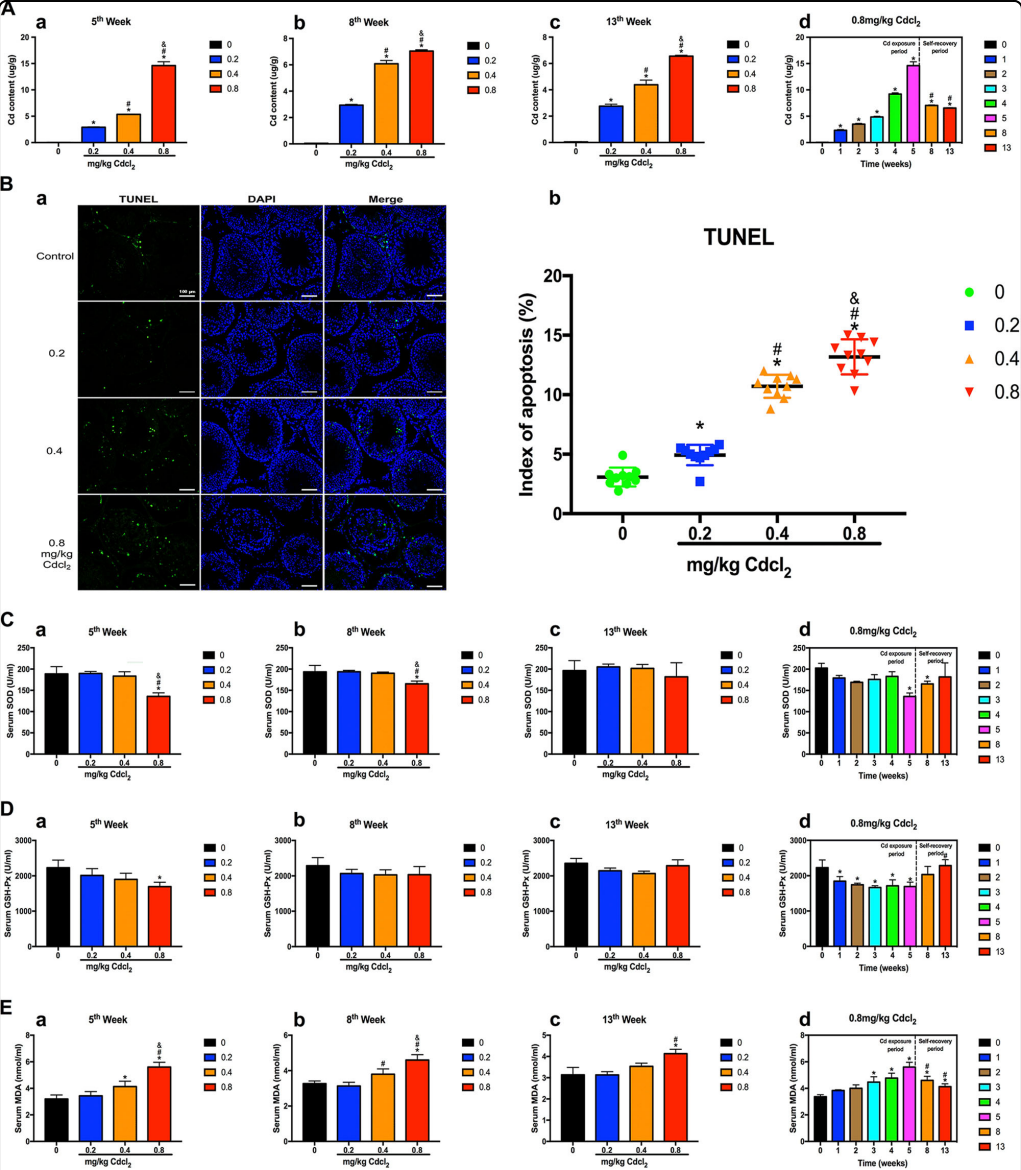
Cd exposure resulted in the continuous accumulation of Cd in the testis and induced testicular cell apoptosis and oxidative stress. **a** Content of Cd in the testis. **b** Effect of Cd on apoptosis in TUNEL-stained sections (200×) at 5th week. Scale bar, 100 μm. **c**–**e** Effects of Cd on oxidative stress: activity of SOD, GSH-Px, and MDA in serum. When it comes to the comparisons of 5th, 8th, and 13th week in 0.8 mg/kg Cd group in **d**, the data is re-used. *P < 0.05 compared with the control. ^#^*P* < 0.05 compared with 5th week. &*P* < 0.05 compared with 8th week.

Cd exposure leaded to accumulation of Cd in the testis (Fig. 2a). Moreover, the number of apoptotic cells was increased in a dose-dependent manner (Fig. 2b). Superoxide dismutase (SOD) and glutathione peroxidase (GSH-Px) activity were decreased and malondialdehyde (MDA) were increased. After self-repair period, SOD and GSH-Px activity returned to normal levels; MDA activity was significantly decreased compared with that at 5th week (Fig. 2c–e). Results suggested that Cd caused the continuous accumulation of Cd in the testis, and induced testicular cell apoptosis and oxidative stress.

**Fig. 3:**
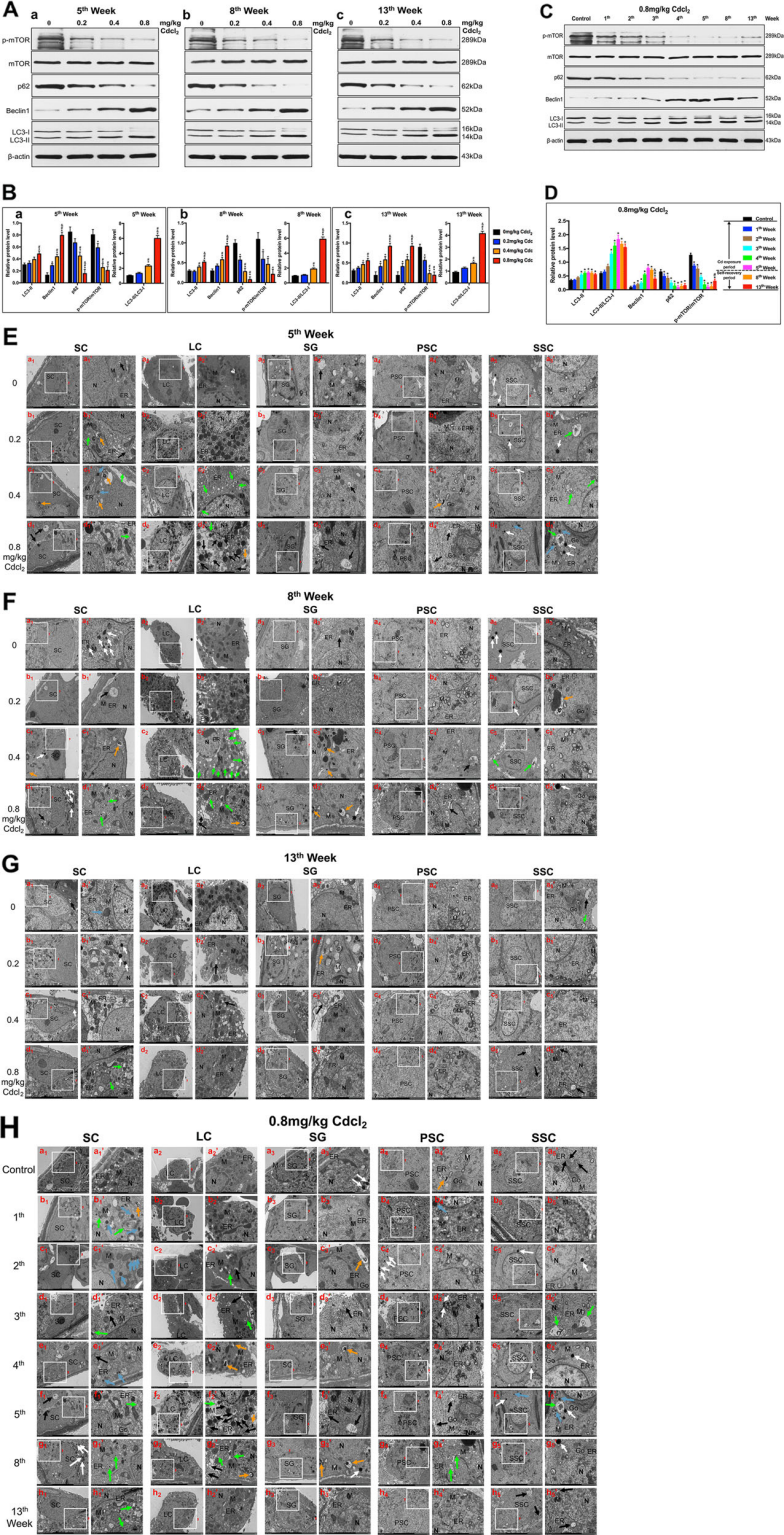
Cd induced testicular autophagy, which was decreased in the self-recovery period. **a**, **b** A representative immunoblot and quantification analysis of LC3, p62, Beclin1, mTOR, and p-mTOR at 5th, 8th, and 13th week. **c**, **d** A representative immunoblot and quantification analysis of LC3, p62, Beclin1, mTOR, and p-mTOR in 0.8 mg/kg Cd group at 1st–13th week. β-actin was used as an internal standard for protein loading. **e**–**h** Representative transmission electron micrographs (TEM) depicting the ultrastructure of SCs, LC, SG, PSC, and SSC in 0.2, 0.4, 0.8 mg/kg Cd group at 5th, 8th, 13th week, and 0.8 mg/kg Cd at 1st–13th week. Areas for the cell in the left column (Scale bar, 5 μm) have been shown for further detail in the right column (Scale bar, 2 μm). SC, Sertoli cells; LC Leydig cells; SG spermatogonium; PSC primary spermatocyte; SSC secondary spermatocyte; N cell nucleus; M mitochondria; ER endoplasmic reticulum; Go Golgi apparatus; Black arrowheads indicate early autophagic vacuoles; green arrowheads indicate typical autophagosomes containing intracellular components and organelles; orange arrowheads indicate late autolysosomes; blue arrowheads indicate lipid droplets; white arrowheads indicate lysosomes. When it comes to the comparisons of 5th, 8th, 13th week in 0.8 mg/kg Cd group in **d** and **h**, the data is re-used. *P < 0.05 compared with the control. ^#^*P* < 0.05 compared with 5th week. ^&^*P* < 0.05 compared with 8th week.

Additionally, we found that autophagy in a time dose-dependent manner was augmented in the Cd-exposure period, whereas lessened in self-recovery period. Cd elevated the expression of positive autophagy-associated proteins (LC3-II and Beclin1), while diminished the expression of negative autophagy-associated proteins (p62), and p-mTOR in a time dose-dependent manner during Cd exposure period (Fig. 3a, b). The expression of the above proteins reached the peak or nadir at 5th week (Fig. 3c, d). Interestingly, after the self-recovery period, the protein expression of LC3-II and Beclin1, LC3-II/LC3-I, p62, and p-mTOR were partially reversed (Fig. 3a–d).

Transmission electron microscopy (TEM) imaging is the gold standard for assessing autophagy. As illustrated in Fig. 3e, Cd stimulated the formation of autophagosomes as well as autophagolysosomes in Sertoli cells (SCs), phagosomes and autophagosomes in Leydig cells (LCs), phagosomes in spermatogonium (SG) and primary spermatocytes (PSCs), lysosomes and lipid droplets in secondary spermatocytes (SSCs). During the self-recovery period, mitochondrial edema, cytoplasm vacuolization, and autophagy only occurred in SCs and LCs in middle/high-dose groups at 8th week (Fig. 3f); a small amount of autophagy sporadically appeared in SCs and SSCs at 13th week (Fig. 3g). At the same dose (0.8 mg/kg), mitochondria and endoplasmic reticulum exhibited progressive aggravation of lesions during 1st–5th week; after stopping Cd exposure, these organelles showed significant improvement at 8th, 13th week. Cd stimulated a wide range of autophagy in testicular cells, including SCs, LCs, SGs, PSCs, and SSCs. Nevertheless, during the self-repair period, autophagy was significantly reduced and mainly occurred in SCs and LCs (Fig. 3h).

Above results indicated that autophagy and apoptosis presented synchronous change trends in Cd-induced testicular injury/self-recovery.

### PI3K inhibitor 3-MA rescued apoptosis by partially aggravating the reduction of autophagy flux in Cd-treated GC-1 cells

**Fig. 4:**
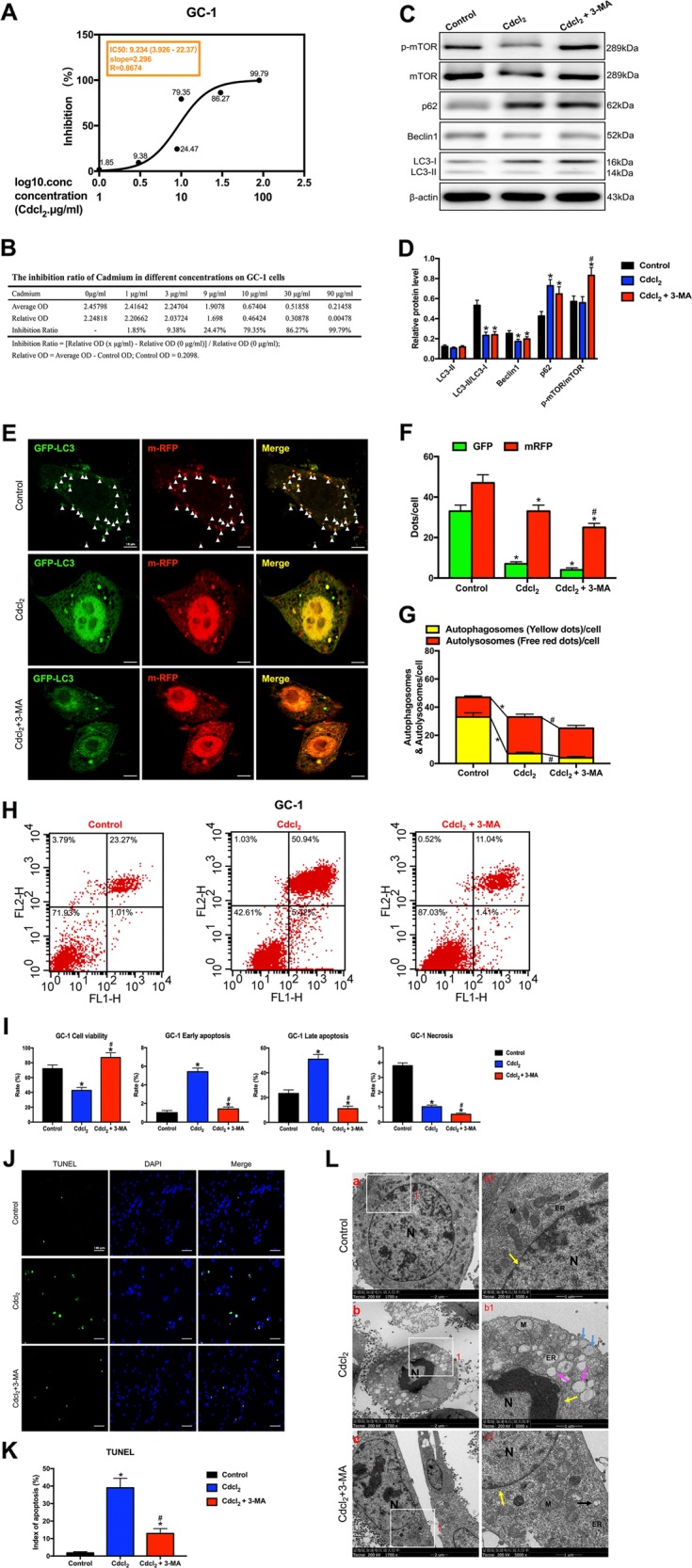
Cd inhibited autophagy and induced apoptosis in GC-1 cells, and the PI3K inhibitor 3-MA protected against Cd-induced GC-1 cells apoptosis by partially aggravating the reduction of autophagic flow with mTOR-independent signaling. **a** The IC50 of Cd for GC-1 cells. **b** The inhibition ratio of Cd at different concentrations on GC-1 cells. **c**, **d** A representative immunoblot and quantification analysis of LC3, p62, Beclin1, mTOR, and p-mTOR in GC-1 cells with or without 3-MA (60 μM) for 24 h. β-actin was used as an internal standard for protein loading. **e** GC-1 cells were transduced with Ad-tf-LC3 for 24 h and were subjected to Cd with 3-MA or without 3-MA for 24 h. Representative images of fluorescent LC3 puncta are shown. **f** Mean number of GFP and mRFP dots per cell. **g** Mean number of autophagosomes (dots with both red and green color, i.e., dots with yellow color in merged images) and autolysosomes (dots with only red and not green color, i.e., dots with red color in merged images) per cell. Adenovirus was transduced at 5 MOI. The results represent the means from at least three independent experiments. Scale bar, 10 μm. **h** Apoptosis of GC-1 cells was detected by flow cytometry. **i** Bar graphs illustrate the percentage of viable cells (Q3), early apoptosis (Q4), late apoptosis (Q2), and necrosis (Q1) as measured by Annexin V/PI staining. **j** The effect of Cd with 3-MA or without 3-MA on apoptosis of TUNEL-stained sections (200×). TUNEL-positive cells were stained and indicated by bright green fluorescence, which were deemed apoptotic cells, and normal nuclei were blue. Scale bar, 100 μm. **k** Quantification of TUNEL detection. **l** The effect of Cd with or without 3-MA on the ultrastructure of GC-1 cells was detected by transmission electron microscopy. Area 1 for the cell in the left column (Scale bar, 2 μm) have been shown for further detail in the right column (Scale bar, 1 μm). N cell nucleus; M mitochondria; ER endoplasmic reticulum; black arrowheads indicate early autophagic vacuoles; green arrowheads indicate typical autophagosomes containing intracellular components and organelles; orange arrowheads indicate late autolysosomes; blue arrowheads indicate lipid droplets; yellow arrowheads indicate nuclear membranes. *P < 0.05 compared with the control. ^#^*P* < 0.05 compared with the Cd-treated group. N.S. not significant.

To further investigate the cross-talk between apoptosis and autophagy, we exerted PI3K inhibitor 3-MA, which was well known as an autophagy inhibitor^6,7^. Initially, we got the half maximal inhibitory concentration (IC50) of Cd for GC-1 cells by the concentration gradient method. The cell counting kit-8 (CCK-8) assay detected the corresponding inhibition rate (1.85, 9.38, 24.47, 79.35, 86.27, and 99.79%) of GC-1 cells after treatment with Cd at different concentrations (1, 3, 9, 10, 30, and 90 μg/ml, respectively) for 24 h (Fig. 4b). Consequently, for subsequent experiments, we determined that the IC50 of Cd for GC-1 cells was 9.234 μg/ml (Fig. 4a, b).

After Cd treatment, the levels of LC3-I and p62 were markedly increased, while the ratio of LC3-II/LC3-I and Beclin1 expression were significantly reduced. However, the PI3K inhibitor 3-MA did not reverse the changes (Fig. 4c, d). To further evaluate autophagic dynamic processes (autophagy flux), which refers to the formation of autophages, the transport of autophagic substrates to lysosomes and the whole process of degradation in lysosomes, we generated an adenovirus harboring tandem fluorescent mRFP-GFP-LC3 (Ad-tf-LC3). GC-1 cells were transduced with Ad-tf-LC3 for 24 h and were then treated with 9.234 μg/ml Cd with or without 3-MA (60 μM) for 24 h. We evaluated the extent of autophagosome and autolysosome formation by using fluorescent LC3 puncta. Cd significantly decreased green and red puncta, and red dots were further decreased in Cd with 3-MA group (Fig. 4e, f). In the merged images, 3-MA significantly aggravated decreases in yellow dots and reversed increases in free red dots induced by Cd, indicating that 3-MA aggravated Cd-induced decreases in autophagosome formation and reversed Cd-induced increases in autolysosome formation (Fig. 4g). These results suggested that 3MA partially aggravated the reduction of Cd-induced autophagy flux*.*

Flow cytometry showed that Cd significantly decreased the percentage of viable cells and increased the percentage of early and late apoptotic cells in the Cd-treated group. Remarkably, 3-MA reversed Cd-triggered GC-1 cell apoptosis (Fig. 4h, i). This result was further confirmed by a TUNEL staining assay. Cd increased the number of apoptotic GC-1 cells, but 3-MA alleviated apoptosis (Fig. 4j, k).

Transmission electron microscopy (TEM) showed that the nuclear membrane structure was complete, and the cytoplasm was enriched with normal organelles in the control group (Fig. 4l(a)). By contrast, Cd-treated group exhibited cell shrinkage and nuclear chromatin condensation with a typical crescent shape, which were markers of apoptosis body (Fig. 4l(b)). Moreover, we observed a ruptured nuclear membrane, increased vacuolization, fewer intracellular organelles and a large number of lipid droplets (Fig. 4l(b1)). Stirringly, 3-MA alleviated the injury (Fig. 4l(c-c1)). Results demonstrated that 3-MA rescued apoptosis by partially aggravating the reduction of autophagy flux in Cd-treated GC-1 cells.

### PI3K inhibitor 3-MA rescued apoptosis by inhibiting autophagy in Cd-treated GC-2/TM3/TM4 cells with mTOR-independent signaling

**Fig. 5:**
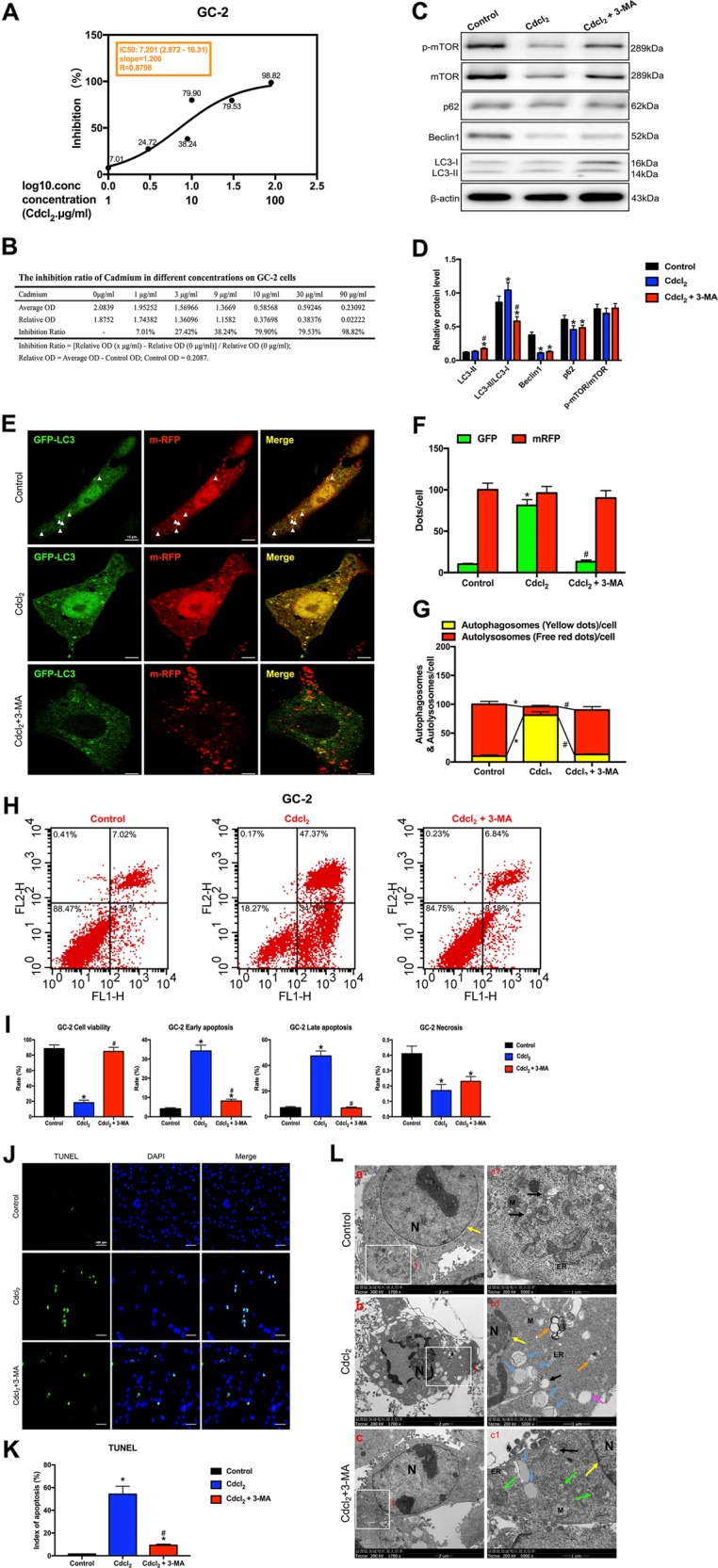
PI3K inhibitor 3-MA rescued apoptosis by inhibiting autophagy in Cd-treated GC-2 cells with mTOR-independent signaling. **a** The IC50 of Cd for GC-2 cells. **b** The inhibition ratio of Cd at different concentrations on GC-2 cells. **c**, **d** A representative immunoblot and quantification analysis of LC3, p62, Beclin1, mTOR, and p-mTOR in GC-2 cells with or without 3-MA (60 μM) for 24 h. β-actin was used as an internal standard for protein loading. **e** GC-2 cells were transduced with Ad-tf-LC3 for 24 h and were subjected to Cd with 3-MA or without 3-MA for 24 h. Representative images of fluorescent LC3 puncta are shown. **f** Mean number of GFP and mRFP dots per cell. **g** Mean number of autophagosomes (dots with both red and green color, i.e., dots with yellow color in merged images) and autolysosomes (dots with only red and not green color, i.e., dots with red color in merged images) per cell. Adenovirus was transduced at 5 MOI. The results represent the means from at least three independent experiments. Scale bar, 10 μm. **h** Apoptosis of GC-2 cells was detected by flow cytometry. **i** Bar graphs illustrate the percentage of viable cells (Q3), early apoptosis (Q4), late apoptosis (Q2), and necrosis (Q1) as measured by Annexin V/PI staining. **j** The effect of Cd with 3-MA or without 3-MA on apoptosis of TUNEL-stained sections (200×). TUNEL-positive cells were stained and indicated by bright green fluorescence, which were deemed apoptotic cells, and normal nuclei were blue. Scale bar, 100 μm. **k** Quantification of TUNEL detection. **l** The effect of Cd with or without 3-MA on the ultrastructure of GC-2 cells was detected by transmission electron microscopy. Area 1 for the cell in the left column (Scale bar, 2 μm) have been shown for further detail in the right column (Scale bar, 1 μm). N cell nucleus; M mitochondria; ER, endoplasmic reticulum; black arrowheads indicate early autophagic vacuoles; green arrowheads indicate typical autophagosomes containing intracellular components and organelles; orange arrowheads indicate late autolysosomes; blue arrowheads indicate lipid droplets; yellow arrowheads indicate nuclear membranes. *P < 0.05 compared with the control. ^#^*P* < 0.05 compared with the Cd-treated group. N.S. not significant.

**Fig. 6:**
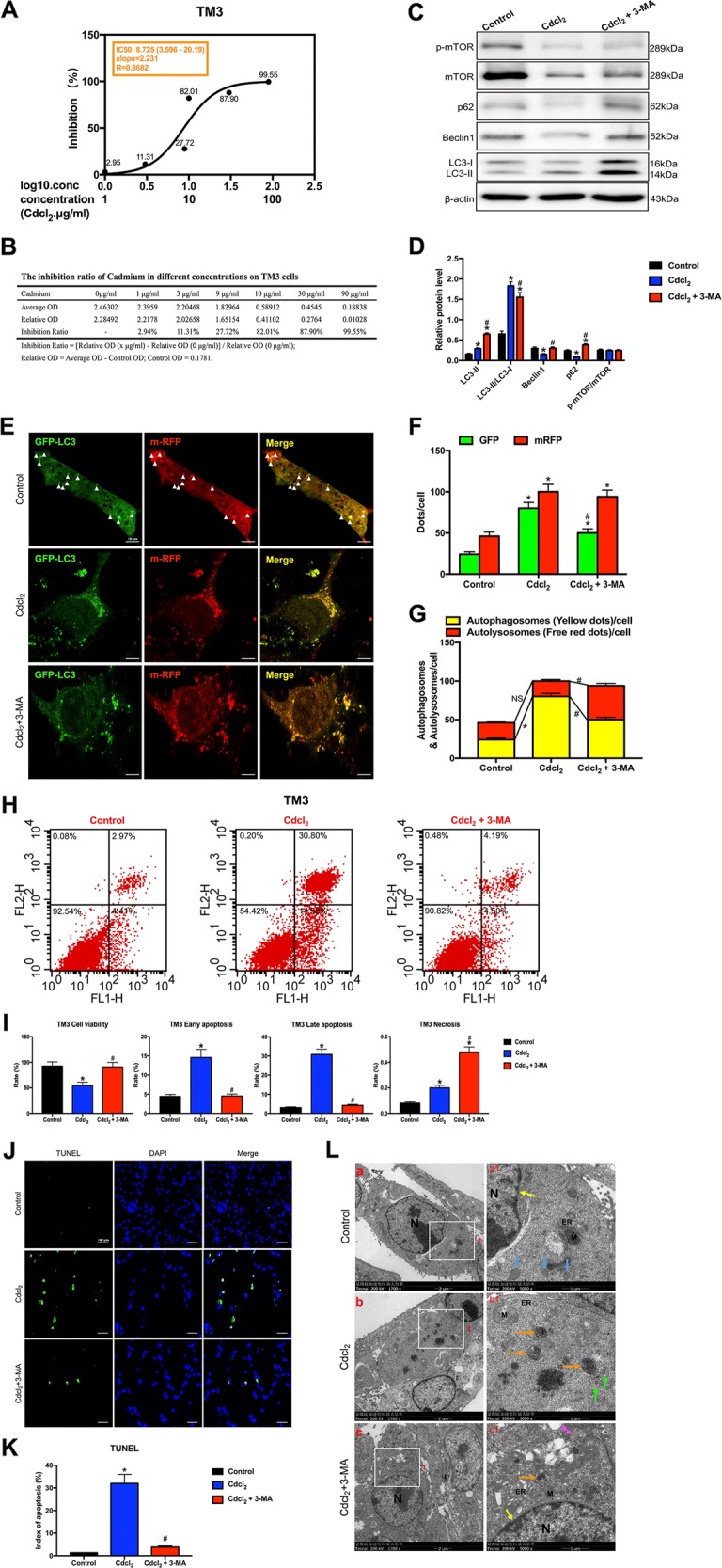
PI3K inhibitor 3-MA rescued apoptosis by inhibiting autophagy in Cd-treated TM3 cells with mTOR-independent signaling. **a** The IC50 of Cd for TM3 cells. **b** The inhibition ratio of Cd at different concentrations on TM3 cells. **c**, **d** A representative immunoblot and quantification analysis of LC3, p62, Beclin1, mTOR, and p-mTOR in TM3 cells with or without 3-MA (60 μM) for 24 h. β-actin was used as an internal standard for protein loading. **e** TM3 cells were transduced with Ad-tf-LC3 for 24 h and were subjected to Cd with 3-MA or without 3-MA for 24 h. Representative images of fluorescent LC3 puncta are shown. **f** Mean number of GFP and mRFP dots per cell. **g** Mean number of autophagosomes (dots with both red and green color, i.e., dots with yellow color in merged images) and autolysosomes (dots with only red and not green color, i.e., dots with red color in merged images) per cell. Adenovirus was transduced at 5 MOI. The results represent the means from at least three independent experiments. Scale bar, 10 μm. **h** Apoptosis of TM3 cells was detected by flow cytometry. **i** Bar graphs illustrate the percentage of viable cells (Q3), early apoptosis (Q4), late apoptosis (Q2), and necrosis (Q1) as measured by Annexin V/PI staining. **j** The effect of Cd with or without 3-MA on apoptosis of TUNEL-stained sections (200×). TUNEL-positive cells were stained and indicated by bright green fluorescence, which were deemed apoptotic cells, and normal nuclei were blue. Scale bar, 100 μm. **k** Quantification of TUNEL detection. **l** The effect of Cd with or without 3-MA on the ultrastructure of TM3 cells was detected by transmission electron microscopy. Area 1 for the cell in the left column (Scale bar, 2 μm) have been shown for further detail in the right column (Scale bar, 1 μm). N cell nucleus; M mitochondria; ER endoplasmic reticulum; black arrowheads indicate early autophagic vacuoles; green arrowheads indicate typical autophagosomes containing intracellular components and organelles; orange arrowheads indicate late autolysosomes; blue arrowheads indicate lipid droplets; yellow arrowheads indicate nuclear membranes. *P < 0.05 compared with the control. ^#^*P* < 0.05 compared with the Cd-treated group. N.S., not significant.

**Fig. 7:**
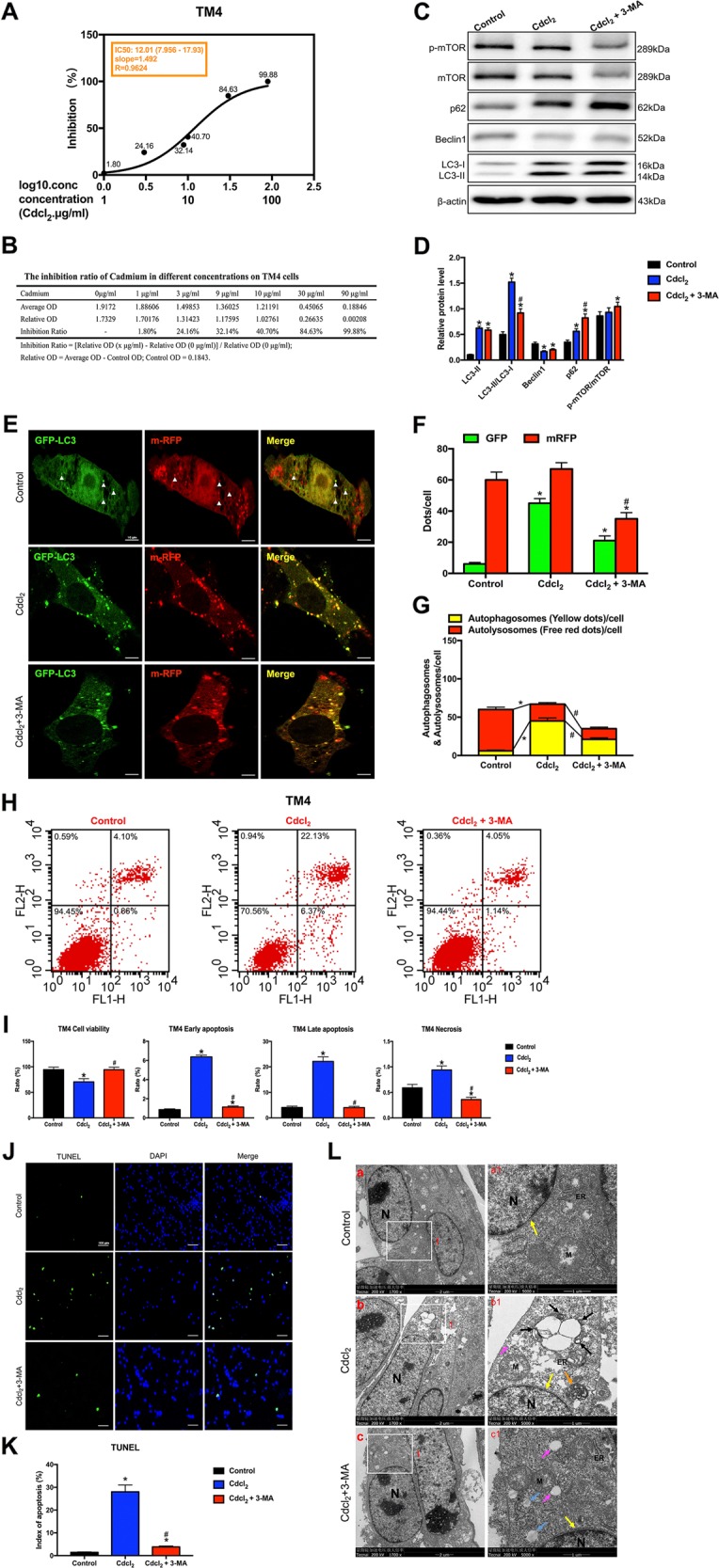
PI3K inhibitor 3-MA rescued apoptosis by inhibiting autophagy in Cd-treated TM4 cells with mTOR-independent signaling. **a** The IC50 of Cd for TM4 cells. **b** The inhibition ratio of Cd at different concentrations on TM4 cells. **c**, **d** A representative immunoblot and quantification analysis of LC3, p62, Beclin1, mTOR, and p-mTOR in TM4 cells with or without 3-MA (60 μM) for 24 h. β-actin was used as an internal standard for protein loading. **e** TM4 cells were transduced with Ad-tf-LC3 for 24 h and were subjected to Cd with 3-MA or without 3-MA for 24 h. Representative images of fluorescent LC3 puncta are shown. **f** Mean number of GFP and mRFP dots per cell. **g** Mean number of autophagosomes (dots with both red and green color, i.e., dots with yellow color in merged images) and autolysosomes (dots with only red and not green color, i.e., dots with red color in merged images) per cell. Adenovirus was transduced at 5 MOI. The results represent the means from at least three independent experiments. Scale bar, 10 μm. **h** Apoptosis of TM4 cells was detected by flow cytometry. **i** Bar graphs illustrate the percentage of viable cells (Q3), early apoptosis (Q4), late apoptosis (Q2), and necrosis (Q1) as measured by Annexin V/PI staining. **j** The effect of Cd with 3-MA or without 3-MA on apoptosis of TUNEL-stained sections (200×). TUNEL-positive cells were stained and indicated by bright green fluorescence, which were deemed apoptotic cells, and normal nuclei were blue. Scale bar, 100 μm. **k** Quantification of TUNEL detection. **l** The effect of Cd with or without 3-MA on the ultrastructure of TM4 cells was detected by transmission electron microscopy. Area 1 for the cell in the left column (Scale bar, 2 μm) have been shown for further detail in the right column (Scale bar, 1 μm). N cell nucleus; M mitochondria; ER endoplasmic reticulum; black arrowheads indicate early autophagic vacuoles; green arrowheads indicate typical autophagosomes containing intracellular components and organelles; orange arrowheads indicate late autolysosomes; blue arrowheads indicate lipid droplets; yellow arrowheads indicate nuclear membranes. *P < 0.05 compared with the control. ^#^*P* < 0.05 compared with the Cd-treated group. N.S. not significant.

Initially, we investigated that IC50 of Cd for GC-2 cells was 7.201 μg/ml, TM3 cells was 8.725 μg/ml, and TM4 cells was 12.01 μg/ml (Figs. 5–7a, b). After Cd treatment, the ratio of LC3-II/LC3-I was significantly increased and subsequently reversed by 3-MA in almost all GC-2/TM3/TM4 cells. Interestingly, the levels of Beclin1 was markedly reduced in almost all Cd-treated GC-2/TM3/TM4 cells, but 3-MA didn’t affect Beclin1. Synchronously, Cd reduced the expression of p62 in GC-2/TM3 cells, but induced the expression of p62 in TM4 cells. Notably, 3-MA has completely different effects on p62 in these three cells lines (Figs. 5–7c, d).

To further evaluate autophagic dynamic processes (autophagy flux), we found that Cd significantly increased green puncta, but green dots were evidently decreased in Cd with 3-MA group in GC-2 cells (Fig. 5e, f). In the merged images, 3-MA significantly reversed Cd-induced increases in yellow dots and decreases in free red dots, indicating that 3-MA antagonized Cd-induced increases in autophagosome formation and decreases in autolysosome formation (Fig. 5g).

In TM3 cells model, Cd significantly increased red and green puncta, but green dots were evidently decreased in Cd with 3-MA group (Fig. 6e, f). In the merged images, 3-MA significantly reversed Cd-induced increases in yellow dots and elevated the number of free red dots, indicating that 3-MA antagonized Cd-induced increases in autophagosome formation (Fig. 6g).

In TM4 cells model, Cd significantly increased green puncta, but green and red dots were evidently decreased in Cd with 3-MA droup (Fig. 7e, f). In the merged images, 3-MA significantly reversed Cd-induced increases in yellow dots and reduced the number of free red dots, indicating that 3-MA antagonized Cd-induced increases in autophagosome formation and aggravated Cd-induced decreases in autolysosome formation (Fig. 7g). These results suggested that 3-MA partially reversed the autophagy flux induced by Cd in GC-2/TM3/TM4 cells.

Flow cytometry indicated that Cd significantly decreased the percentage of viable cells and increased the percentage of early and late apoptotic cells in the Cd-treated group (Figs. 5–7h, i). TUNEL staining assay further confirmed that 3-MA alleviated GC-2/TM3/TM4 cells apoptosis induced by Cd (Figs. 5–7j, k).

TEM showed that Cd induced severe cell shrinkage, chromatin condensation, and fragmentation, especially mature autophagolysosomes and large lipid droplets in GC-2 cells (Fig. 5l(a, b)); cells nuclei disappeared and mature autophagolysosomes per cell were markedly increased in Cd-treated TM3 cells (Fig. 6l(a, b)); large vacuoles, early and late autophagolysosomes showed in Cd-treated TM4 cells (Fig. 7l(a, b)). Whereas 3-MA obviously alleviated Cd-induced autophagic death in GC-2/TM3/TM4 cells (Figs. 5–7l(c-c1)).

As mTOR signaling played a key role in driving autophagy, we next examined whether it was involved in Cd-induced testicular cells injury/recovery. Results showed that Cd didn’t change the ratios of Ser2448p-mTOR/mTOR protein expression in all four testicular cells, while 3-MA activated mTOR signaling only in GC-1 and TM4 cells. Given that, we demonstrated that the regulation of autophagy is not dependent on mTOR signaling in Cd-treated testicular cells lines (Figs. 5–7c, d).

Above results suggested that 3-MA protected against Cd-induced GC-2/TM3/TM4 cells apoptosis by inhibiting autophagy with mTOR-independent signaling.

## Discussion

**Fig. 8:**
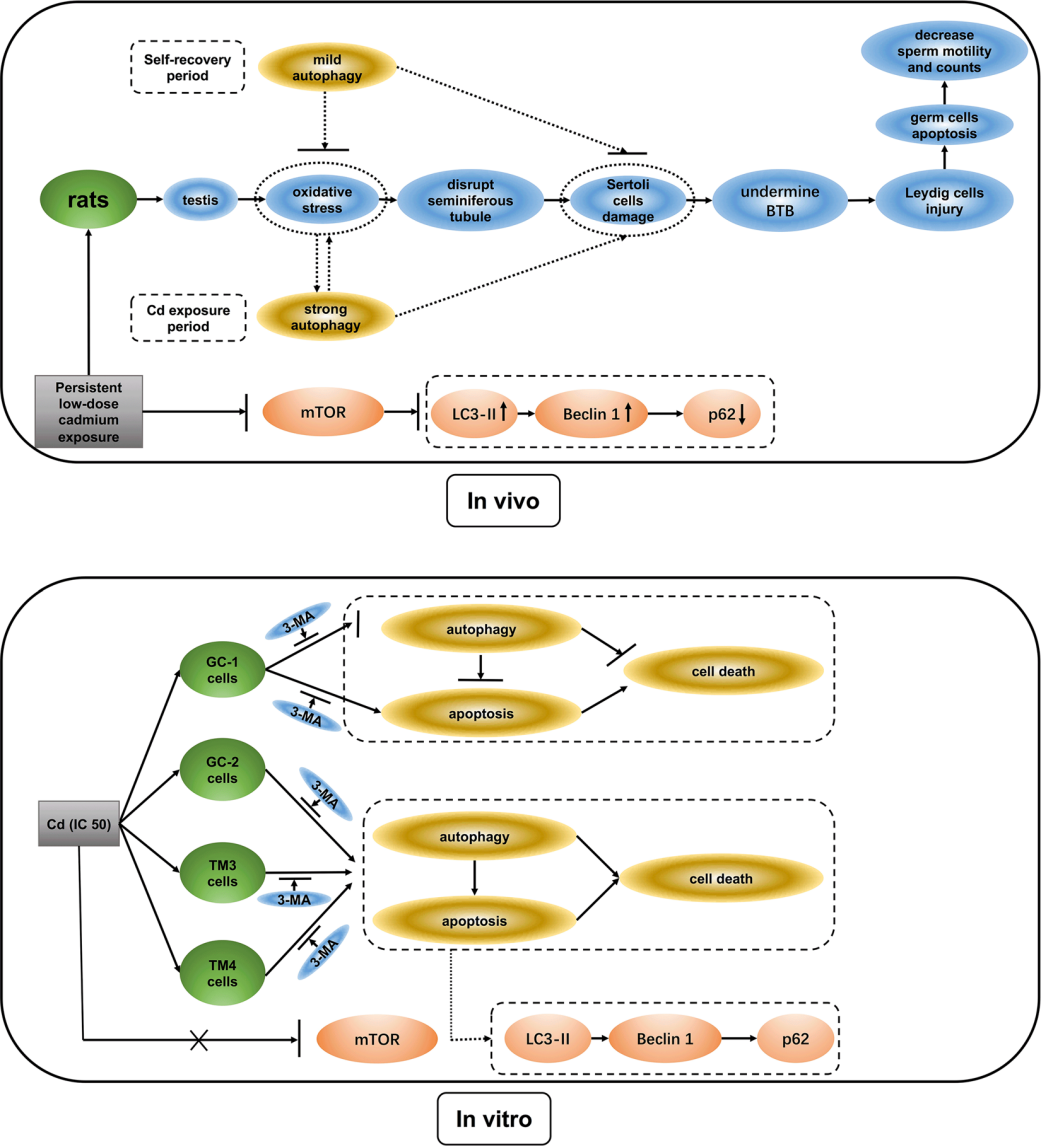
Conclusions: Diagram tying together the cross-talk of autophagy and apoptosis in Cd-induced injury/recovery of testicular cells in vivo and vitro. In vivo, persistent low-dose Cd exposure induces male rat reproductive lesions via eliciting oxidative stress, damaging the morphology of seminiferous tubule, lowering testicular marker enzymes activity in Sertoli cell and germ cell, and decreasing sperm motility and counts. Meanwhile, Cd induces autophagy and apoptosis in testicular cells via restraining mTOR pathway in rats. After one cycle of spermatogenesis 8 weeks without Cd exposure, some reproductive injury has recovered, which indicates that the testis has some self-repair function. In reparative phase, autophagy is significantly diminished. These results suggest that this may be due to the fact that strong autophagy promotes apoptosis and kills cells during Cd exposure period, while mild autophagy antagonizes apoptosis and protects cells during self-recovery period. In vitro, Cd-induced GC-2/TM3/TM4 cells autophagic apoptosis could be rescued by PI3K inhibitor 3-MA inhibiting autophagy; but Cd inhibits autophagy and induces apoptosis in GC-1 cells, and 3-MA protects against Cd-induced GC-1 cell apoptosis by inhibiting autophagy flux. However, mTOR signaling was not linked with GC-1/GC-2/TM3/TM4 cells autophagy. Together, we provide the relationships among autophagy, apoptosis, Cd exposure, spermatogenesis dysfunction, and infertility. These results suggest that cross-talk between autophagy and apoptosis regulates testicular injury/recovery induced by cadmium via PI3K with mTOR-independent pathway, especially in SCs and LC.

The current study demonstrates that low-concentration persistent Cd exposure induces male reproductive toxicity, and first evidences that testes have a certain self-repair ability, and the cross-talk of autophagy and apoptosis regulates testicular injury/recovery induced by Cd with mTOR-independent pathway (Fig. 8). We establish a chronic animal model to mimic environmental Cd exposure on human, and report that autophagy and apoptosis show a synergistic trend in Cd-induced testicular injury/self-recovery. In parallel, Gump et al. also demonstrates that strong autophagy promotes apoptosis and kills cells, while mild autophagy antagonizes apoptosis and protects cells^14^. Thus, all results suggest that a potential similar cell death mechanism about autophagy and apoptosis exists in testicular cells. Given that, we utilize PI3K inhibitor 3-MA, also known as an autophagy inhibitor^12,13^, to explore the cross-talk of autophagy and apoptosis in four testicular cells (GC-1/GC-2/TM3/TM4 cells) respectively.

In vivo study, Cd exposure for five consecutive weeks resulted in the continuous accumulation of Cd in the testes, which affected the body weight and organ coefficient. Cd induced oxidative stress and lowered the activity of testicular marker enzymes. Then, the morphology of testicular tissue was damaged, resulting in a significant decrease in sperm count and motility. After one cycle of spermatogenesis and 8 weeks without Cd exposure (i.e., at the end of 13th week), above toxic effects were ameliorated.

Testicular marker enzymes include LDH, AKP, ACP, and γ-GT. Among these enzymes, LDH indicates energy supply level in the development and maturation of germ cell^15^; AKP is linked with the division of germ cells for the energy transport of glucose; ACP mainly exists in the cytoplasm of SCs, and functions on the degeneration of seminiferous epithelium^16^;γ-GT plays a role in SCs and promotes sperm maturation^17^. In our study, Cd abated the activity of LDH, AKP, ACP, and γ-GT, manifesting that Cd damaged testicular cells, including germ cells and SCs. Actually, sperm motility and counts are the most important determinants of male fertility^18^. Cd dwindled sperm counts and motility, whereas sperm counts were picked up and sperm motility didn’t rise again during the self-repair period. As secretions from the prostate are necessary for sperm motility^19^, this finding is consistent with the result of the prostate organ coefficient. Then, how is the sperm damaged in chronic Cd exposure?

Previously, our research corroborated that oxidative stress played a crucial role in acute Cd exposure^20^. Oxidative stress represents an imbalanced status between oxidants and antioxidants^21^. Although sperm production requires physiological levels of oxides, excessive oxides produced by environmental toxicants can have a detrimental effect, even leading to the apoptosis of germ cells and LCs. This hypothesis is in agreement with a previous study by Mathur et al.^22^. Here, we found that SOD and GSH-Px (antioxidant enzymes) activities were lower, and MDA (an indicator of peroxidation) level was significantly higher in 0.8 mg/kg Cd-treated rats. Since normal active antioxidant enzymes can effectively scavenge free radicals and promote the development and differentiation of germ cells, abnormal activity may lead to spermatogenesis disorders^23^. What is the fundamental mechanism of spermatogenesis disruption induced by Cd?

We found that the levels of positive autophagy-associated proteins LC3-II and Beclin1 were significantly elevated, and the level of negative autophagy-associated protein p62 was reduced during the Cd exposure period. Ultrastructural observation showed that Cd induced early autophagic vacuoles, autophagosomes, and autolysosomes in rat testicular cells, especially in SCs and LCs. During the self-repair period, autophagy evidently declined. Results revealed that the testis may have a certain metabolic capacity for Cd during the self-repair period. This capacity may be linked with the interaction between autophagy and apoptosis.

To our knowledge, autophagy and apoptosis are key to controlling cell death. Autophagy is a double-edged sword: it can contribute to cell survival by removing damaged proteins, organelles, pathogens or aggregates;^24^ conversely, autophagy can also promote cell death through excessive self-digestion and degradation of essential cellular components^14,25^. Determining exactly when and where these disparate functions of autophagy apply are momentous goals in the field.

In vitro study, we exploited four testicular cell lines to verify the underlying mechanism of cross-talk between autophagy and apoptosis. Ad-tf-LC3 is a useful tool for evaluating the formation of both autophagosomes and autolysosomes simultaneously^26^. PI3K inhibitor 3-MA, is also known as an autophagy inhibitor^12,13^. Remarkably, 3-MA reversed Cd-induced autophagy flux changes via enhancing autophagosomes conversion to autolysosomes, as indicated by a decrease in autophagosomes and an increase in autolysosomes in GC-2 cells; 3-MA reversed autolysosomes formation in Cd-treated GC-1 and GC-2 cells, and autophagosomes formation in GC-2/TM3/TM4cells (Figs. 5–7g). 3-MA permanently inhibits type I PI3K but transiently impedes type III PI3K, thus mainly inhibiting autophagosome formation^27^. This finding may explain why 3-MA alleviated LC3-II accumulation induced by Cd and significantly inhibited autophagosome formation in GC-2/TM3/TM4 cells. Instead, Cd inhibited autophagy in GC-1 cells. These results provide further evidences that the effects of 3-MA on the dynamic process of autophagy varies from different testicular cells, but 3-MA indeed inhibits autophagy in Cd-treated testicular cells.

As we know, mTOR is a central checkpoint that negatively regulates autophagy^28,29^. Of note, mTOR is vital upstream of autophagy during oxidative stress^30^. Therefore, we suspected that the oxidative stress-mediated mTOR signaling pathway may be the main way to increase the potential autophagy and induce the reproductive toxicity of Cd. In our animal study, Cd dephosphorylated mTOR in the testis. However, in vitro study, Cd did not influence the mTOR signaling in GC-1/GC-2/TM3/TM4 cells. Interestingly, 3-MA activated mTOR signaling in GC-1 and TM4 cells. Moreover, increases in cell apoptosis and autophagy and decreases in cell viability induced by Cd were rescued by 3-MA. Collectively, these results suggest that mTOR signaling may not be linked to Cd-induced autophagy and apoptosis in vitro. Based on our current data, it is reasonable to speculate that an intricate endocrine mechanism is involved in Cd-induced autophagic cell death in the testis^31^, which may be related to mTOR signaling suppression. In contrast, at the cellular level of vitro, there is relatively different homeostasis in testicular cell autophagic death. Thus, mTOR signaling was not affected by Cd in vitro. In our next study, we will perform specific experiments designed to address this aspect of our observations.

Generally, the interplay between autophagy and apoptosis is complex^32^. Apoptosis and autophagy promote or antagonize each other, and the two processes occur independently^33^. Several studies have reported that autophagy is a trigger for cell death and that autophagy activity contributes to PCD in Caenorhabditis elegans^34,35^. Similarly, our results revealed that Cd could induce apoptosis and diminish cell viability in GC-1/GC-2/TM3/TM4 cells simultaneously, as indicated by increases in the index of apoptosis by TUNEL, the apoptosis rate by flow cytometry, as well as changes in cellular ultrastructure. Interestingly, 3-MA effectively reversed Cd-induced apoptosis and ultrastructural injury in these four testicular cell lines. Conclusively, 3-MA protected against Cd-induced GC-2/TM3/TM4 cell apoptosis by inhibiting autophagy. Notably, in GC-1 cells, Cd inhibited autophagy and induced apoptosis, while 3-MA rescued apoptosis by partially aggravating the reduction of autophagy flux. Interestingly, mTOR signaling wasn’t influenced by Cd in four testicular cells, although 3-MA activated mTOR signaling in GC-1/TM4 cells.

Overall (Fig. 8), the most striking finding of this study is that strong autophagy promotes apoptosis and kills cells in Cd exposure period; whereas mild autophagy antagonizes apoptosis and protects cells in self-recovery period of the testis. This is also the first evidence that cross-talk between autophagy and apoptosis regulates testicular injury/recovery induced by Cd via PI3K with mTOR-independent pathway*,* especially in SCs and LC. In fact, cell death/survival depends on a complex network of molecular interactions between apoptosis and autophagy^36,37^. However, the precise mechanism of regulating autophagy and apoptosis remains to be illustrated. Consequently, the cross-talk and feedback mechanisms between apoptosis and autophagy are worthy of further investigation.

## Methods

### Ethics statement

All the animal procedures were approved by the Institutional Animal Care and Use Committee of Tongji Medical College, Huazhong University of Science and Technology. All experiments with rats were conducted ethically according to the Guide for the Care and Use of Laboratory Animal guidelines.

### Chemicals and reagents

Cd chloride and ascorbic acid were purchased from Sigma Chemical Co. (St. Louis, MO, USA). SOD, MDA, GSH-Px, LDH, AKP, and ACP assay kits were provided by Nanjing Jiancheng Bioengineering Institute (Nanjing, China). γ-GT was obtained from Changchun Huili Biotech CO., LTD (Changchun, China). A bicinchoninic acid (BCA) protein assay kit and enhanced chemiluminescence (ECL) kit were purchased from Beyotime Institute of Biotechnology (Shanghai, China). Monoclonal antibodies were partly purchased from Cell Signaling Technology (Cambridge, MA, USA), including mTOR and p-mTOR (Ser2448) antibodies (CST, Cat#2972, Cat#2971). p62, LC3B, and Beclin1 antibodies were obtained from Santa Cruz Biotechnology (Santa Cruz, Cat#sc-48402, Cat#sc-398822, Cat#sc-48341). 3-MA was purchased from MedChemExpress (MCE, Cat#HHY-19312, USA). CCK-8 was purchased from Dojindo (Kumamoto, Japan). An Annexin V-Fluorescein isothiocyanate (FITC) and Propidium Iodide (PI) Detection Kit was purchased from BD Biosciences (New Jersey, USA). A TUNEL kit was purchased from F. Hoffmann-La Roche (Basel, Switzerland). Dulbecco’s Modified Eagle’s Medium (DMEM) was purchased from HyClone (Logan, Utah, USA). Collagenase and fetal bovine serum (FBS) were purchased from Gibco (Australia). mRFP-GFP-LC3 adenoviral particles were purchased from Vigene Bioscience Inc (Shandong, China).

### Animals and cell lines

Eight-weeks-old adult male Sprague-Dawley (SD) rats (230 ± 30 g) were supplied by Tongji Medical College Animal Center (Wuhan, China). All procedures were performed in accordance with the Guide for the Care and Use of Laboratory Animals published by the Ministry of Health of People’s Republic of China. Animals were allowed to adapt to the new environment for 3 days and were given a standard diet and water ad libitum. The conditions were controlled as follows: temperature (22–26 °C), humidity (50 ± 5%) and a 12-h light/dark cycle. Four testicular cells lines (GC-1 cells: mouse spermatogonial cells; GC-2 cells: mouse spermatocyte cells; TM3 cells: mouse leydig cells; TM4 cells: mouse Sertoli cells) were obtained from the Institute of Reproductive Health, Tongji Medical College and were tested for mycoplasma contamination.

### Experimental design

#### Animal model

Four groups (Group 1–4) were designed in the study. Group 1 was treated with 0.9% NaCl. Groups 2, 3, and 4 were treated with 0.2, 0.4, and 0.8 mg/kg CdCl_2_, respectively. CdCl_2_ was dissolved in 0.9% NaCl. The intraperitoneal injection volume was adjusted according to the weight of each rat. All groups were treated between 8:30 and 11:30 a.m. every day for five consecutive weeks. Approximately 102 healthy male rats (weight 230 ± 30 g) in this study were randomly divided into 17 endpoints. The number of examination endpoints in each group was different (shown in Supplementary Fig. 5A). Four endpoints in Group 1 (0.9% NaCl) were designated on day 1 and weeks 5, 8, and 13. Three endpoints in Group 2 (0.4 mg/kg Cdcl_2_) and Group 3 (0.4 mg/kg Cdcl_2_) were on week 5, 8, and 13. Seven endpoints in Group 4 (0.8 mg/kg Cdcl_2_) were on week 1, 2, 3, 4, 5, 8, and 13. Consequently, there were 17 endpoints in total. The timing was determined by the spermatogenetic cycle and sperm transit time in rats.

#### Cell model

Four male reproductive cell lines (GC-1, GC-2, TM3, and TM4 cells) were respectively cultured in DMEM supplemented with 10% FBS at 37 °C in a humidified atmosphere containing 5% CO_2_ and 95% air. 3-Methyladenine (3-MA) is an inhibitor of PI3K. It is a widely used inhibitor of autophagy via its inhibitory effect on class III PI3K^12,13^. To establish the Cd model, we treated each cell line with the IC50 Cd without 3-MA or with 3-MA (60 μM) for 24 h. Investigators and data analyst were blind to the group allocation.

### Body weights and organ coefficients

At 24 h after the last treatment, rats were weighed. Blood samples were drawn from the heart to measure several biochemical markers. Then, the animals were sacrificed by decapitation. The testis, epididymis, liver, spleen, kidney, seminal vesicle, prostate, and paranephros were removed immediately and weighed. Parts of the testis and other organs were fixed with Bouin’s fixative and 4% paraformaldehyde for paraffin-embedded sectioning and histopathological analysis, respectively, and others were frozen in liquid nitrogen for Western blotting.

### Sperm parameter analysis

Basic semen analysis followed the recommendations of the World Health Organization (WHO) 2010 manual for the examination of human semen (WHO, 2010). The sperm concentration was assessed using an improved Neubauer hemocytometer, and the total sperm count was calculated by multiplying the semen volume and concentration. Motility measurements were performed to record the progressive sperm count per 200 sperm under a microscope.

### Testicular marker enzyme activities

The left testis was homogenized, and homogenized mixture was centrifuged in a Kubota 6030 centrifuge (Kubota Corp., Tokyo, Japan). The supernatant was used for enzyme assays. Reagent kits for LDH, AKP and ACP were purchased from Nanjing Jiancheng Bioengineering Institute (Nanjing, China), and γ-GT was purchased from Changchun Huili Biotech CO., LTD (China). Enzyme activities were measured according to the manufacturer’s specifications.

### Histopathological analyses of the testis

Hematoxylin and eosin staining is the most commonly used technique in histology and routine pathology. Testis sections were embedded in paraffin, stained with hematoxylin and eosin (HE), and examined under a light microscope for observation of structural abnormalities.

### Cd content in the testis

Cd content in testis was detected by graphite furnace atomic absorption spectrometry (GFAAS). The testicular tissue was baked and weighed. We added nitric acid and perchloric acid to dissolve into a salt free from flowing liquid on a 280 °C hotplate for 1 h. After the samples cooled, 5 ml pure water was added to volume. The operating conditions for GFAAS require atomization at 1600 °C for Cd after a heating phase (110 °C and then 1308 °C) and pyrolysis at 500 °C for Cd. The wavelength was 228.80 nm for Cd.

### Assessment of apoptosis by TUNEL staining

DNA fragmentation in apoptotic cells was detected using a TUNEL assay kit. Briefly, testis sections were embedded in paraffin, cut into 5-µm-thick sections, and incubated with the TUNEL kit. Cells were fixed in 4% paraformaldehyde solution for 20 min at room temperature. The cells were counterstained with DAPI to mark the nuclei and were then visualized by fluorescence microscopy. Apoptotic cell nuclei appeared green, and overlapping green fluorescence and blue nuclear fluorescence indicated apoptosis.

### Serum biochemical assay

After standing for 30 min, the blood was centrifuged for 10 min at 3000 rpm to collect serum. Samples were stored at −20 °C and measured as soon as possible. The levels of SOD, MDA, and GSH-PX in serum were determined according to the manufacturer’s instructions.

### Western blotting

Rats were sacrificed via decapitation, and the proteins in the testes were immediately extracted. The protein concentration in the extracts was determined using a BCA protein assay kit (Beyotime Institute of Biotechnology, China). Proteins were denatured, separated on sodium dodecyl sulfate-polyacrylamide gel electrophoresis (SDS-PAGE) gels, and transferred to a polyvinylidene fluoride (PVDF) membrane. Then, the membranes were incubated with antibodies according to the manufacturer’s instructions, including mTOR/p-mTOR, LC3B, p62, and Beclin1. Next, the membranes were rinsed and incubated with horseradish peroxidase (HRP)-conjugated secondary antibodies. The target proteins were detected with ECL (Beyotime Institute of Biotechnology, China). The density was analyzed by Quantity One Software (Bio-Rad, Hercules, CA).

### Transmission electron microscopy

Testes, GC-1/GC-2/TM3/TM4 cells were respectively fixed with 2.5% glutaraldehyde for 2 h at 4 °C, postfixed in 1% osmium tetroxide, and embedded in Epon 812. Blocks were cut into semithin sections and stained with methanolic uranyl acetate and lead citrate. The testicular ultrastructure was investigated by TEM (JEM 1200-EX; Hitachi, Ltd, Tokyo, Japan) at 80 kV, and the ultrastructure of four cells was investigated by TEM (Tecnai G2 20 TWIN; FEI, Hillsboro, OR) at 200 kV.

### CCK-8 assay

Cell viability was assessed with a CCK-8 assay according to the manufacturer’s instructions. Cells were plated in 96-well plates at a density of 10,000 cells/plate. After the indicated treatments were completed, the culture medium was replaced with 110 μl CCK-8 solution (containing 100 μl serum-free DMEM and 10 μl CCK-8 reagent). After the cells were incubated for 4 h, we measured the absorbance in each well at a wavelength of 450 nm with a microplate spectrophotometer.

### mRFP-GFP-LC3 adenovirus transduction and confocal microscopy

mRFP-GFP-LC3 adenoviral particles were purchased from Vigene Bioscience Inc (Shandong, China). Cells were infected with adenoviral particles at 5 multiplicity of infection (MOI). At 24 h after adenovirus transduction, the cells were washed and fixed with 4% paraformaldehyde. Confocal sections were collected with a Nikon A1 laser scanning confocal microscope (Nikon America Inc., Melville, NY) under uniform settings. The number of GFP and mRFP dots was determined by manual counting of fluorescent puncta from at least four different cell preparations with a 100× objective.

### Flow cytometry analysis

To analyze the effects of the indicated treatments on cell survival, we stained the cells with an Annexin V-FITC and PI Detection Kit and analyzed them by flow cytometry. Flow cytometry data were assessed using BD FACSDiva Software v7.0 (Becton-Dickinson, USA).

### Statistical analysis

The data were expressed as the mean ± S.D. Differences among groups were analyzed by the Student’s *t*-test, one-way analysis of variance (ANOVA), or Mann–Whitney *U*-test. A difference was considered significant when *P* < 0.05. The showed experiments were replicated 4–6 times in the laboratory.

## Supplementary information

Supplementary Figure 1
