# Supplementary figures and images for: Cross-talk between autophagy and apoptosis regulates testicular injury/recovery induced by cadmium via PI3K with mTOR-independent pathway
Source: Cell Death Dis. 2020 Jan 22;11(1):46. doi: 10.1038/s41419-020-2246-1 (PMC6976559; doi:10.1038/s41419-020-2246-1)

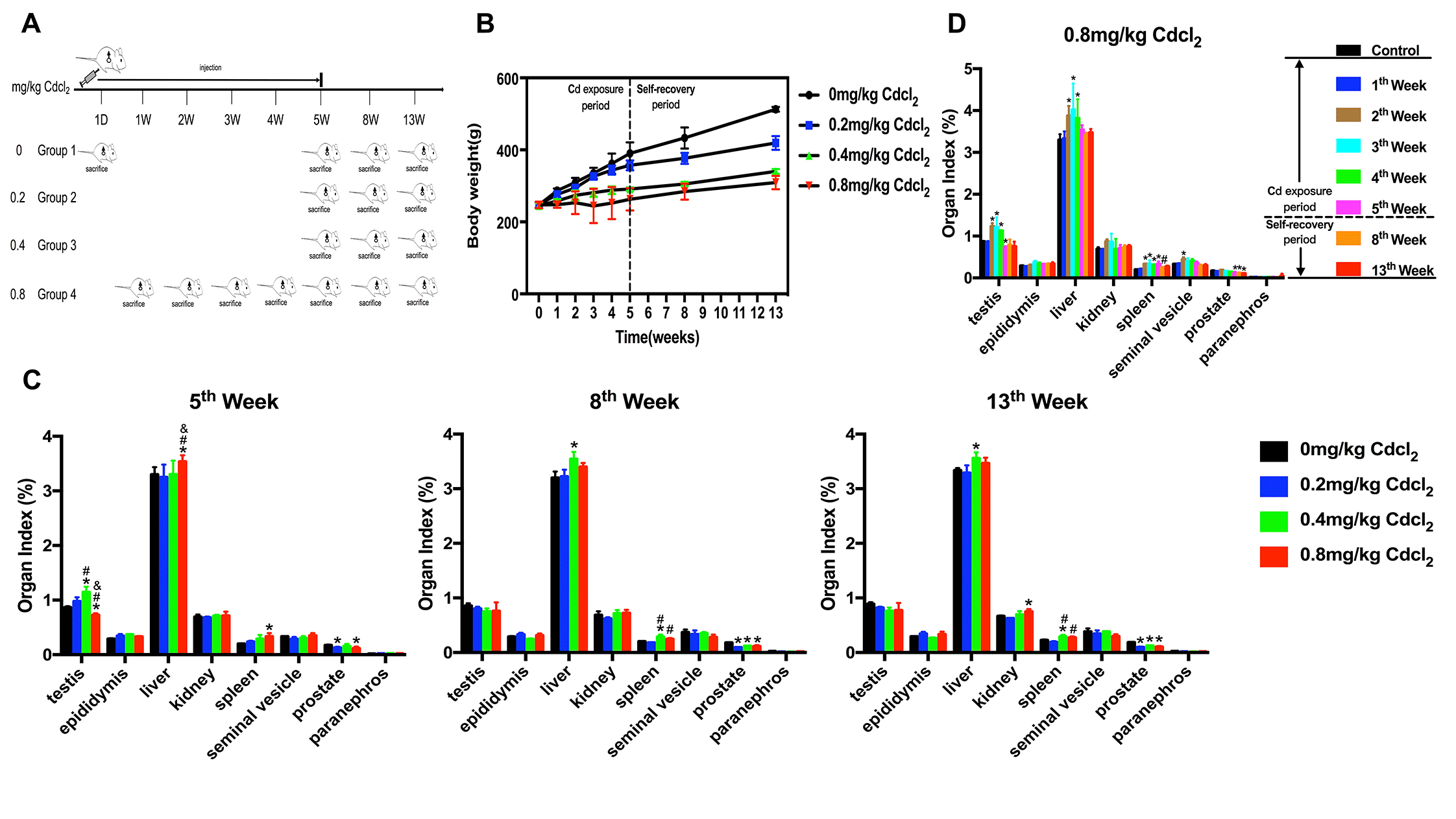

Supplement: Supplementary file 1 — Supplementary Figure 1 [file 41419_2020_2246_MOESM1_ESM.png]
